# Tumor-Infiltrating Immune-Related Long Non-Coding RNAs Indicate Prognoses and Response to PD-1 Blockade in Head and Neck Squamous Cell Carcinoma

**DOI:** 10.3389/fimmu.2021.692079

**Published:** 2021-10-19

**Authors:** Ben Ma, Hongyi Jiang, Yi Luo, Tian Liao, Weibo Xu, Xiao Wang, Chuanpeng Dong, Qinghai Ji, Yu Wang

**Affiliations:** ^1^ Department of Head and Neck Surgery, Fudan University Shanghai Cancer Center, Shanghai, China; ^2^ Department of Oncology, Shanghai Medical College, Fudan University, Shanghai, China; ^3^ Center for Computational Biology and Bioinformatics, Indiana University School of Medicine, Indianapolis, IN, United States; ^4^ Department of Biohealth Informatics, School of Informatics and Computing, Indiana University, Indianapolis, IN, United States

**Keywords:** HNSCC, Ti-lncRNA, PD-1 blockade, CTLA4, prognosis

## Abstract

Long non-coding RNAs (lncRNAs) in immune cells play critical roles in tumor cell–immune cell interactions. This study aimed to characterize the landscape of tumor-infiltrating immune-related lncRNAs (Ti-lncRNAs) and reveal their correlations with prognoses and immunotherapy response in head and neck squamous cell carcinoma (HNSCC). We developed a computational model to identify Ti-lncRNAs in HNSCC and analyzed their associations with clinicopathological features, molecular alterations, and immunotherapy response. A signature of nine Ti-lncRNAs demonstrated an independent prognostic factor for both overall survival and disease-free survival among the cohorts from Fudan University Shanghai Cancer Center, The Cancer Genome Atlas, GSE41613, and GSE42743. The Ti-lncRNA signature scores in immune cells showed significant associations with *TP53* mutation, *CDKN2A* mutation, and hypoxia. Inferior signature scores were enriched in patients with high levels of PDCD1 and CTLA4 and high expanded immune gene signature (IGS) scores, who displayed good response to PD-1 blockade in HNSCC. Consistently, superior clinical response emerged in melanoma patients with low signature scores undergoing anti-PD-1 therapy. Moreover, the Ti-lncRNA signature was a prognostic factor independent of PDCD1, CTLA4, and the expanded IGS score. In conclusion, tumor-infiltrating immune profiling identified a prognostic Ti-lncRNA signature indicative of clinical response to PD-1 blockade in HNSCC.

## Introduction

Head and neck squamous cell carcinoma (HNSCC) originates from epithelial cells at sites of oral cavity, pharynx, and larynx, which is the sixth most common cancer worldwide, with 890,000 new cases and 450,000 deaths in 2018 (1, 2). Several common risk factors for HNSCC have been uncovered, such as smoking, alcohol abuse, consumption of areca catechu, human papillomavirus (HPV) infection, and exposure to environmental pollutants (3, 4). Surgery, radiation, and systemic therapy are the principal modalities for locally confined HNSCC. A majority of the patients with recurrent or metastatic HNSCC are considered for systemic therapy, especially immunotherapy, except for some patients cured by local management (5, 6).

In general, the tumor microenvironment (TME) of HNSCC is highly infiltrated by immune cells with regard to tumor biology, which mediate immune surveillance or evasion through various mechanisms (3). In advanced-staged HNSCC, it is demonstrated that the cytotoxic activities of T cells are repressed due to the upregulation of immunosuppressive factors such as PD-1 and CTLA4 in TME, leading to persistent efforts of reactivating T cells to treat this malignancy (7–10). Until now, immune checkpoint inhibitors have significantly updated the therapeutic modalities of HNSCC. The Food and Drug Administration approved the use of the immune checkpoint inhibitors pembrolizumab and nivolumab for the treatment of cisplatin-refractory recurrent or metastatic HNSCC and pembrolizumab as a first-line therapy for unresectable or metastatic disease in 2016 and 2019, respectively (9–11). However, it is noted that only a subset of patients are expected to respond to immune checkpoint inhibitors and that reliable predictive biomarkers are needed.

Therefore, it is necessary to identify molecular biomarkers that can be used to predict the disease progression, survival status, and response to immunotherapy of HNSCC. The search for such biomarkers has focused on the molecular abnormalities of tumor-infiltrating immune cells. In recent years, long non-coding RNAs (lncRNAs) in immune cells have demonstrated to play critical roles in tumor cell–immune cell interactions (12, 13). In the present study, we initially characterized the lncRNA landscape of immune cells specifically altered in HNSCC and then aimed to identify a prognostic lncRNA signature that is useful for the prediction of immunotherapy response through integrated analyses of tumor-infiltrating immune-related lncRNAs (Ti-lncRNAs) and clinicopathological features.

## Materials and Methods

### Transcriptional Data of Immune Cells and Tumor Cell Lines

The transcriptional profiles of 115 purified cell lines of 19 immune cell types based on the Affymetrix HG-U133_Plus 2.0 platform were obtained from the Gene Expression Omnibus (GEO) database (http://www.ncbi.nlm.nih.gov/geo), including GSE13906 (14), GSE23371 (15), GSE25320 (16), GSE27291 (17, 18), GSE27838 (19), GSE28490 (20), GSE28698 (21), GSE28726 (22), GSE37750 (23), GSE39889 (24), GSE42058 (25), GSE49910 (26), GSE51540 (27), GSE59237 (28), GSE6863 (29), and GSE8059 (30). We obtained the transcriptional profiles of HNSCC cell lines based on the Affymetrix HG-U133_Plus 2.0 platform from the Cancer Cell Line Encyclopedia (CCLE) project (https://portals.broadinstitute.org/ccle) and collected 34 cell lines that matched the tumor type ‘HNSC’ from the cell annotation files of The Cancer Genome Atlas (TCGA).

### Microarray Data Processing

All raw data (.cel files) of the 115 immune cells and the 34 ‘HNSC’ cell line microarray data profiled by the Affymetrix HG-U133_Plus 2.0 platform were downloaded and processed together using robust multi-array average (RMA) normalization with the R ‘affy’ packages. RMA normalization for the patient datasets GSE41613 and GSE42743 (31) was performed separately as well. The Affymetrix Human Genome U133 Plus 2.0 Array probes were re-annotated into unique Ensembl gene IDs using custom library file downloaded from the Brainarray database (HGU133Plus2_Hs_GENCODEG, version 24; http://mbni.org/customcdf/24.0.0/gencodeg.download/HGU133Plus2_Hs_GENCODEG_24.0.0.zip). In total, 54,675 probes were mapped to 21,311 ensemble genes, including 3,599 genes that were annotated as lncRNAs in the GENCODE annotation file (version 32, http://ftp.ebi.ac.uk/pub/databases/gencode/Gencode_human/release_32/gencode.v32.annotation.gtf.gz). The 3,477 lncRNAs that existed in both the microarray and TCGA profiles were selected for subsequent analysis.

### Transcriptional Profiles of HNSCC Patients

HNSCC patients with transcriptional profiles were obtained from the GEO database, the TCGA database (https://portal.gdc.cancer.gov), and The cBioPortal for Cancer Genomics (32) (http://www.cbioportal.org/) according to the following selection criteria: 1) with detailed information of stage, age, gender, and overall survival (OS) time and status; 2) profiled with the Affymetrix HG-U133_Plus 2.0 or Illumina HiSeq platform; and 3) sample size large than 50. In total, 671 HNSCC patients were enrolled, including two microarray profiles, GSE41613 (*n* = 97) and GSE42743 (*n* = 74), and TCGA dataset (*n* = 500). TCGA dataset was used as the training dataset for discovering a lncRNA signature; the other two microarray datasets were used as independent test datasets for validating the lncRNA signature. Detailed clinical information of the three patient sets is shown in **Table 1**.

**Table 1:** Clinical characteristics of patients with head and neck squamous cell carcinoma (HNSCC) enrolled in this study.

Variables	TCGA (training)		TCGA (test)		FUSCC		GSE41613		GSE42743	
	*N*	%	*N*	%	*N*	%	*N*	%	*N*	%
Age (years)										
≤60	142	47.3	101	50.5	41	51.25	50	51.5	40	54.1
>60	158	52.7	99	49.5	39	48.75	47	48.5	34	45.9
Gender										
Male	216	72.0	151	75.5	70	87.5	66	68.0	58	78.4
Female	84	28.0	49	24.5	10	12.5	31	32.0	16	21.6
TNM stage										
I–II	67	22.3	47	23.5	25	31.25	41	42.3	30	40.5
III–IV	227	75.7	145	72.5	51	63.75	56	57.7	44	59.5
Unknown	6	2.0	8	4.0	4	5.0	0	0.0	0	0.0
Survival status										
Alive	169	56.3	112	56.0	55	68.75	46	47.4	32	43.2
Dead	130	43.3	87	43.5	25	31.25	51	52.6	42	56.8
Unknown	1	0.3	1	0.5	0	0.0	0	0.0	0	0.0

TCGA, The Cancer Genomics Atlas; FUSCC, Fudan University Shanghai Cancer Center; TNM, tumor node metastasis.

### Development of a Ti-lncRNA Signature

**Figure 1:**
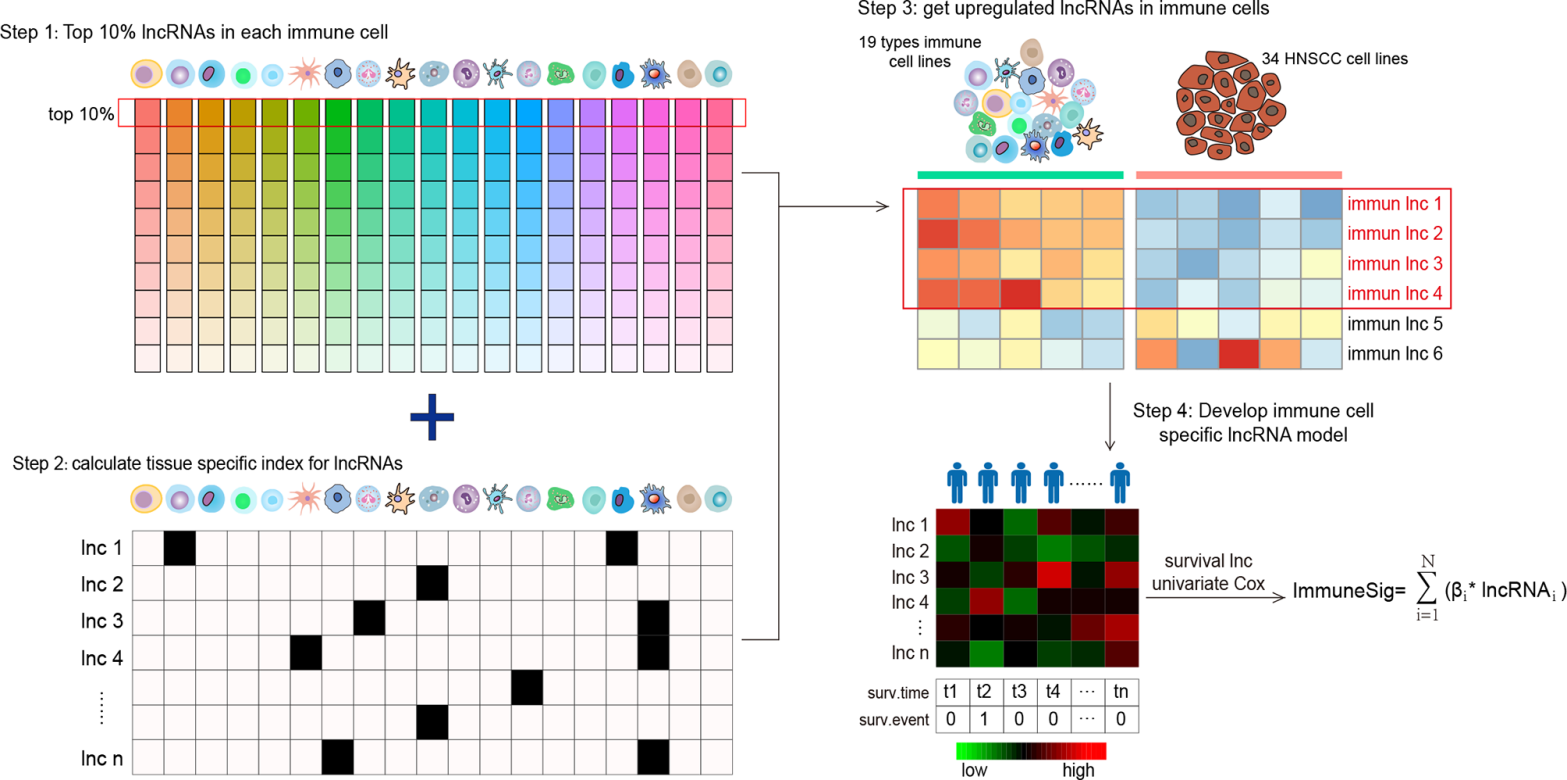
Flow graph for characterizing the lncRNA landscape of HNSCC-infiltrating immune cells and for building a prognostic signature. Step 1: all lncRNAs were ranked according to their mean expression levels in each immune cell type, and the top 10% expressed lncRNAs were chosen as candidate lncRNAs. Step 2: the TSI scores of a total of 958 lncRNAs were calculated to reflect their expression specificity with respect to the 19 immune cell types. Step 3: lncRNAs with high TSI values (> 0.1) were further analyzed for differential expression between the 19 types of immune cells and the 34 HNSCC cell lines. Step 4: the lncRNAs upregulated in immune cells were screened out and compared with HNSCC cells. lncRNA, long non-coding RNA; HNSCC, head and neck squamous cell carcinoma; TSI, tissue specificity index.

We established a novel Ti-lncRNA by integrative lncRNA profiling analyses on purified immune cells, HNSCC cell lines, and cancer bulk tissues as follows (**Figure 1**): 1) the top 10% expressed lncRNAs in each immune cell (average expression value) were obtained for the 19 immune cell types; 2) the immune cell specificity of lncRNA was calculated with the tissue specificity index (TSI) using the following formula:


$$\text{TS}{\text{I}}_{\mathrm{lnc}}=\frac{\sum_{i=1}^{N}\left(1-x_{\mathrm{lnc},i}\right)}{N-1}$$

where *N* denotes the total number of immune cell types and *x*_lnc,_ *_i_* is the expression intensity of immune cells normalized by the maximal expression of any immune cell types for lncRNAs. A higher TSI value represents a higher cell specificity of the lncRNA. TSI ranges from 0 to 1; 3) differential expression analyses were further performed for the top expressed lncRNAs with high TSI values (>0.1) between immune cells and the HNSCC cell line profiles. The lncRNAs with a false discovery rate (FDR) <0.05 upregulated in immune cells were recognized as Ti-lncRNAs; 4) the OS-related Ti-lncRNAs were selected using univariate Cox regression analyses. Finally, nine lncRNAs with a univariate *p*-value <0.01 were selected for the construction of a prognostic risk model using the linear combination of the expression values of the prognostic Ti-lncRNAs, weighted by their estimated regression coefficients from multivariate Cox regression analyses.

### HNSCC Patients from FUSCC

A total of 80 HNSCC patients were enrolled in this study, who received surgical therapy at Fudan University Shanghai Cancer Center (FUSCC) from 2011 to 2019. The 80 samples obtained from these patients, whose diagnoses were confirmed by pathological experts, were subjected to RNA extraction and further lncRNA expression analyses. The clinical features of the 80 patients are described in **Table 1**. Each patient provided written informed consent for his/her specimens and information to be used for research and stored in the hospital database. This study was approved by the Medical Ethics Committee of the FUSCC. All procedures performed in our study were in accordance with the ethical standards of our institutional research committee and with the 1964 Helsinki Declaration and its later amendments or comparable ethical standards.

### RNA Extraction and Expression Analyses of lncRNAs

We extracted total RNA from the HNSCC samples using the TRIzol reagent (Life Technologies, Carlsbad, CA, USA). Total RNA was reverse transcribed to cDNA using the TAKARA PrimeScriptTM RT Master Mix (Perfect Real Time). As previously described (33), for real-time quantitative PCR, the TAKARA TB green was used and the following lncRNAs were employed: ENSG00000265148, ENSG00000281358, ENSG00000262089, ENSG00000240889, ENSG00000253230, ENSG00000261888, ENSG00000235304, ENSG00000226806, and ENSG00000260244. The assays were performed in triplicate for each sample, and the mean value was used for the calculation of the lncRNA expression levels. The relative lncRNA expression levels were determined by the comparative CT (2^−ΔCT^) method. The lncRNA expression levels were given as ratios to the β-actin messenger RNA (mRNA) level. The primer sequences for each lncRNA are attached in **Supplementary Table S1**.

### Hypoxia, Cancer-Associated Fibroblast, and Tumor-Associated Macrophage

The Nurmik mRNA-based cancer-associated fibroblast (CAF) gene signature was used to quantify the CAFs in HNSCC according to previous studies (34, 35). We quantified tumor hypoxia in HNSCC by applying the mRNA-based hypoxia signature from Buffa et al. (36) The signature genes of hypoxia and CAF are shown in **Supplementary Table S1**. A summary score of hypoxia or CAF is defined in each sample as the median of the absolute expression values of the genes in the signature, as described in previous studies (35, 37). The xCell tool (http://xcell.ucsf.edu/) (38) was used to infer the enrichment score of the M2 cell type by using the transcriptome data of TCGA cohort, and M2 was considered as a tumor-associated macrophage (TAM) in our study.

### Gene Set Enrichment Analysis

Gene set enrichment analysis (GSEA) was performed using the GSEA software, version 4.1.0, which was obtained from the Broad Institute (http://www.broad.mit.edu/gsea), as previously described (39, 40). Enrichment Map was used for the visualization of the GSEA results. The normalized enrichment score (NES) and *p*-value were used to sort the pathways enriched in each phenotype after gene set permutations were performed 1,000 times for each analysis.

### Statistical Analysis

Continuous variables were expressed as the mean value ± standard deviation (SD), and categorical data were summarized with frequencies and percentages. Independent *t*-test was used to compare the continuous variables between two groups. *χ^2^* and Fisher’s exact tests were used for categorical variables. The expression levels of the nine lncRNAs as a signature in each patient were integrated into a risk score: −0.724 × ENSG00000265148 + (−1.047) × ENSG00000281358 + (−0.159) × ENSG00000262089 + (−0.887) × ENSG00000240889 + 2.137 × ENSG00000253230 + (−0.656) × ENSG00000261888 + (−0.556) × ENSG00000235304 + (−1.257) × ENSG00000226806 + (−0.195) × ENSG00000260244. To analyze the associations between the lncRNA signature and the clinicopathological parameters, patients were divided into two subgroups (low risk score and high risk score groups) according to the median value of the risk score. Nonparametric receiver operating characteristic (ROC) analyses were performed to calculate the area under the curve (AUC) for the signature that would be predictive of the survival status. Univariate and multivariate logistic regression analyses were performed to determine the risk factors for the signature. The Kaplan–Meier method was used to construct survival curves, and the survival difference was determined by the log-rank test. A *p*-value <0.05 was considered significant. Univariate and multivariate Cox regression methods were utilized to conduct survival analyses. Data preparation and statistical analyses were performed using the SPSS for Windows (version 22.0; IBM Corp., Armonk, NY, USA), the R software (version 3.5.1; R Foundation for Statistical Computing, Vienna, Austria), and GraphPad Prism (version 6.01; GraphPad Software Inc., La Jolla, CA, USA).

## Results

### Identification of Ti-lncRNAs Specifically Altered in Immune Cells of HNSCC

The landscape of lncRNAs was initially characterized in all human immune cells, and a differential expression pattern was observed in 19 immune cell types (**Supplementary Figure S1**). As shown in **Figure 1**, to capture representative lncRNAs in different immune cell types, we firstly ranked all lncRNAs according to the mean expression levels of each immune cell, and the top 10% expressed lncRNAs were chosen as candidate lncRNAs. A total of 958 lncRNAs were selected for the next procedure (**Supplementary Table S2**). Then, we calculated the TSI scores for the 958 immune-related lncRNAs to reflect their expression specificity with respect to the 19 immune cell types. The top 872 expressed lncRNAs with high TSI values (>0.1) were further analyzed for differential expression between the 19 types of immune cells and the 34 HNSCC cell lines. As a result, 492 lncRNAs were identified as Ti-lncRNAs, which were significantly upregulated with FDR < 0.05 in immune cells compared with HNSCC cells (**Supplementary Table S2**).

**Figure 2:**
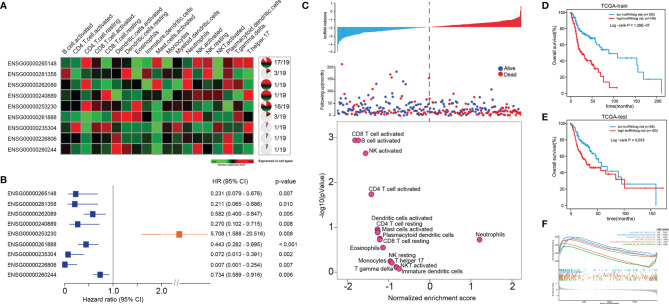
A Ti-lncRNA signature consisting of nine lncRNAs was identified as a prognostic indicator associated with OS in TCGA cohort. **(A)** Expression heatmap (*left*) and expression specificity proportion (*right*) of the nine Ti-lncRNAs in the 19 immune cell types. **(B)** Forest plot of the hazards ratios (HRs) with 95% confidence interval **(CI)** to show the associations of the Ti-lncRNAs with OS in the training cohort of TCGA. **(C)** A Ti-lncRNA signature was developed using a linear combination of the expression values of the nine Ti-LncRNAs, weighted by their estimated regression coefficients from multivariate Cox regression analysis, and volcano plots for the enrichment of immune cell types for patients with high scores and low scores calculated based on the NES from the GSEA. **(D)** Differences in OS between patients with low signature risk scores and those with high risk scores was analyzed in the training cohort of TCGA using the Kaplan–Meier method to construct an OS curve. **(E)** The Ti-lncRNA signature was analyzed for its association with survival in the test cohort of TCGA. **(F)** Biological signaling pathways enriched in patients with high Ti-lncRNA scores. lncRNA, long non-coding RNA; OS, overall survival; TCGA, The Cancer Genome Atlas; Ti-lncRNA, tumor-infiltrating immune-related lncRNA; NES, normalized enrichment score; GSEA, gene set enrichment analysis.

We further sought to identify Ti-lncRNAs associated with HNSCC survival outcomes using univariate Cox regression analyses in the training cohort of TCGA. Patients from TCGA were randomly split into 60% (*n* = 300) and 40% (*n* = 200) as a training cohort and a test cohort, respectively. There were nine Ti-lncRNAs (ENSG00000265148, ENSG00000281358, ENSG00000262089, ENSG00000240889, ENSG00000253230, ENSG00000261888, ENSG00000235304, ENSG00000226806, and ENSG00000260244) with different expression specificities in the 19 immune cell types (**Figure 2A** and **Supplementary Table S3**), which were remarkably correlated with OS in the training cohort (**Figure 2B**).

### The Ti-lncRNA Signature as an Independent Prognostic Factor for HNSCC

We developed a Ti-lncRNA signature score using a linear combination of the expression values of the nine Ti-LncRNAs, which were weighted by their estimated regression coefficients from multivariate Cox regression analysis. The Ti-lncRNA signature was identified as a prognostic indicator associated with OS in the training cohort of TCGA (log-rank *p* < 0.001) (**Figures 2C, D**). As shown in **Figure 2C**, a majority of the immune signaling pathways (16/17, 94.11%) were enriched in patients with low Ti-lncRNA signature scores, while the neutrophil signaling pathway (1/17, 5.89%) was enriched in patients with high risk scores. We confirmed the significant correlation of a high Ti-lncRNA score with a decreased OS time (log-rank *p* = 0.033) (**Figure 2E**) in the test cohort of TCGA. We performed GSEA using the RNA sequencing data of the whole TCGA cohort to investigate the associations of the signature with the tumor signaling pathways. Among all predefined pathway gene sets, the biological pathways Myc targets v2 (NES = 1.86, *p* = 0.010), interferon alpha response (NES = 2.07, *p* = 0.020), TGF-β signaling (NES = 1.68, *p* = 0.009), and glycolysis (NES = 2.07, *p* = 0.043) were enriched in the phenotype with a high risk score (**Figure 2F**).

**Figure 3:**
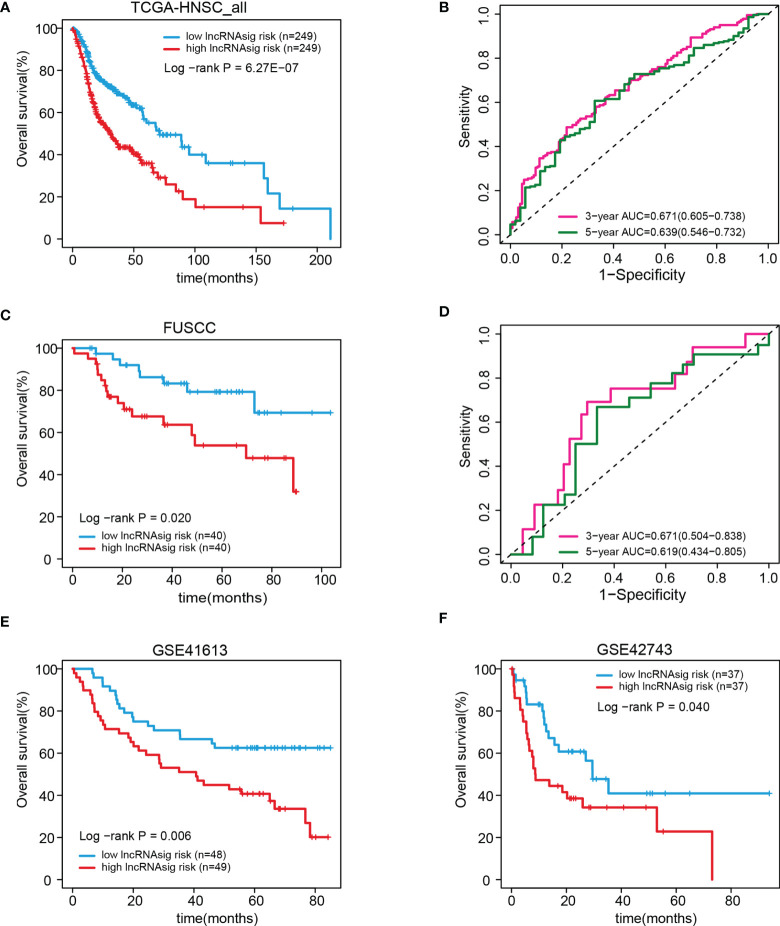
Validation of the prognostic effect of the Ti-lncRNA signature in patients from TCGA, FUSCC, GSE41613, and GSE42743. **(A–F)** Differences in OS and the predictive effect of the signature risk score were analyzed in the whole TCGA cohort **(A, B)**, the FUSCC cohort **(C, D)**, and the GSE41613 and GSE42743 cohorts **(E, F)**. lncRNA, long non-coding RNA; Ti-lncRNA, tumor-infiltrating immune-related lncRNA; TCGA, The Cancer Genome Atlas; FUSCC, Fudan University Shanghai Cancer Center; OS, overall survival.

**Figure 4:**
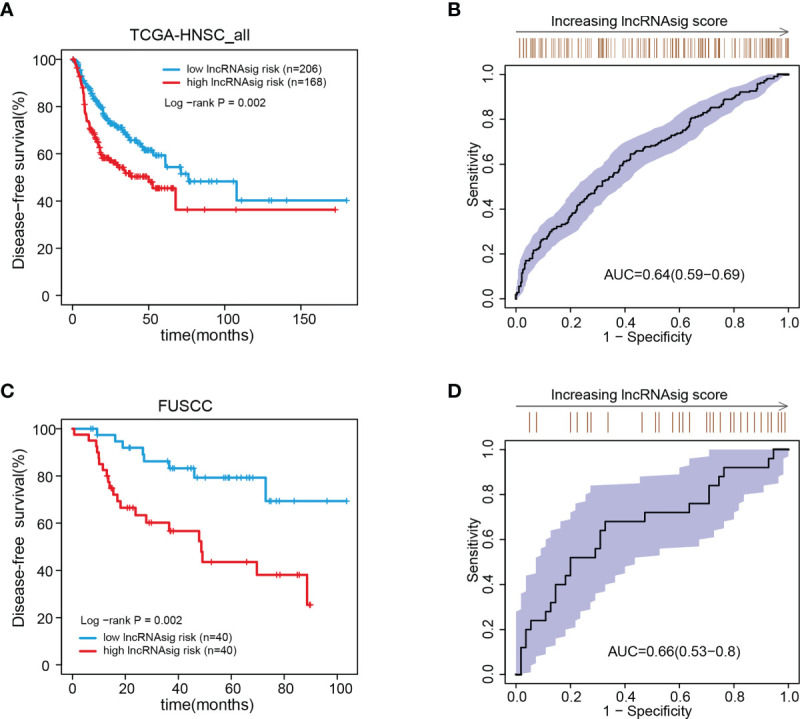
Associations of the Ti-lncRNA signature with DFS in patients from TCGA and FUSCC. **(A–D)** Differences in DFS and the predictive effect of the signature risk score were analyzed in TCGA cohort **(A, B)** and in the FUSCC cohort **(C, D)**. *lncRNA*, long non-coding RNA; Ti-*lncRNA*, tumor-infiltrating immune-related lncRNA; *DFS*, disease-free survival; *TCGA*, The Cancer Genome Atlas; *FUSCC*, Fudan University Shanghai Cancer Center.

We further validated the prognostic effect of the Ti-lncRNA signature in the FUSCC cohort (80 patients) and the public cohorts (TCGA, 498 patients; GSE41613, 97 patients; and GSE42743, 74 patients). The Ti-lncRNA signature demonstrated a significant association with the OS of patients among all four cohorts (TCGA: log-rank *p* < 0.001; FUSCC: log-rank *p* = 0.020; GSE41613: log-rank *p* = 0.006; GSE42743: log-rank *p* = 0.040) (**Figure 3**), and its predictive scores for decease status were proven to be relatively high at 3 years (TCGA: AUC = 0.671; FUSCC: AUC = 0.671) and at 5 years (TCGA: AUC = 0.639; FUSCC: AUC = 0.619) (**Figures 3B, D**, respectively). Additionally, we analyzed the correlations of the Ti-lncRNA signature with recurrence of HNSCC in 80 patients from FUSCC and in 374 patients from TCGA. A high signature score indicated a shortened disease-free survival (DFS) in both cohorts (TCGA: log-rank *p* = 0.0015; FUSCC: log-rank *p* = 0.002), with a relatively high predictive effect for recurrence (FUSCC: AUC = 0.64; TCGA: AUC = 0.66) (**Figure 4**).

Moreover, univariate and multivariate Cox regression analyses were performed to verify the independent effect of the Ti-lncRNA signature on the prognoses of patients. As shown in **Table 2**, after adjusting for age, gender, and tumor node metastasis (TNM) stage, a high signature score was repeatedly recognized as a risk factor for shortened OS time in the FUSCC cohort (multivariate: HR = 2.495, 95%CI = 1.058–5.881, *p* = 0.037), TCGA cohort (HR = 2.214, 95%CI = 1.669–2.939, *p* < 0.001), the GSE41613 cohort (HR = 1.768, 95%CI = 0.980–3.192, *p* = 0.059), and the GSE42743 cohort (HR = 1.911, 95%CI = 1.003–3.641, *p* = 0.049). The Ti-lncRNA signature was an independent risk factor for recurrence in the FUSCC cohort (*p* = 0.010, HR = 3.005, 95%CI = 1.307–6.910) and TCGA cohort (*p* = 0.001, HR = 1.835, 95%CI = 1.299–2.593), as well as in the multivariate Cox regression analyses after adjusting for age, gender, and TNM stage (**Table 3**).

**Table 2:** Univariate and multivariate Cox regression analyses of overall survival in the datasets.

Variables	Univariate analysis			Multivariate analysis		
	HR	95%CI of HR	*p*-value	HR	95%CI of HR	*p*-value
TCGA						
lncRNAsig (high/low)	1.996	1.513–2.633	**<0.001**	2.214	1.669–2.939	**<0.001**
Age	1.021	1.008–1.033	**<0.001**	1.024	1.011–1.037	**<0.001**
Gender (male/female)	0.751	0.563–1.000	0.050	0.798	0.594–1.073	0.136
TNM stage (I/II *vs*. III/IV)	1.216	0.877–1.685	0.242	1.259	0.906–1.748	0.170
GSE41613						
lncRNAsig (high/low)	2.185	1.230–3.882	**0.008**	1.76812	0.980–3.192	**0.059**
Age group^a^	0.924	0.689–1.239	0.598	0.9365	0.685–1.281	0.681
Gender (male/female)	1.123	0.621–2.029	0.702	1.11925	0.618–2.029	0.710
TNM stage (I/II *vs*. III/IV)	3.829	1.959–7.485	**<0.001**	3.36525	1.699–6.666	**<0.001**
GSE42743						
lncRNAsig (high/low)	1.892	1.019–3.512	**0.043**	1.91053	1.003–3.641	**0.049**
Age	1.004	0.980–1.028	0.764	1.013	0.988–1.038	0.305
Gender (male/female)	0.676	0.337–1.355	0.270	0.56938	0.281–1.154	0.118
TNM stage (I/II *vs*. III/IV)	2.917	1.210–7.034	**0.017**	3.09155	1.259–7.590	**0.014**
FUSCC						
lncRNAsig (high/low)	2.636	1.132–6.137	**0.025**	2.4945	1.058–5.881	**0.037**
Age	1.016	0.971–1.063	0.505	1.0137	0.967–1.063	0.574
Gender (male/female)	0.751	0.256–2.205	0.602	0.8999	0.301–2.695	0.851
TNM stage (I/II *vs*. III/IV)	2.276	0.777–6.667	0.134	2.3862	0.801–7.107	0.118

HR, hazard ratio; CI, confidence interval; lncRNAsig, long non-coding RNA signature; TCGA, The Cancer Genomics Atlas; TNM, tumor node metastasis; FUSCC, Fudan University Shanghai Cancer Center.

a Age groups: 19–39 years, 40–49 years, 50–59 years, and 60–68 years.

Bold type indicates statistical significance.

**Table 3:** Univariate and multivariate Cox regression analyses of disease-free survival in the datasets.

Variable	Univariate analysis			Multivariate analysis		
	HR	95%CI of HR	*p*-value	HR	95%CI of HR	*p*-value
TCGA						
lncRNAsig (high/low)	1.707	1.221–2.385	**<0.001**	1.835	1.299–2.593	**0.001**
Age	1.012	0.996–1.028	**<0.001**	1.017	1.000–1.034	0.051
Gender (male/female)	0.959	0.654–1.406	0.829	0.967	0.649–1.442	0.871
TNM stage (I/II *vs*. III/IV)	1.369	0.884–2.119	0.159	1.428	0.918–2.222	0.114
FUSCC						
lncRNAsig (high/low)	3.336	1.472–7.562	**0.004**	3.005	1.307–6.910	**0.010**
Age	1.015	0.974–1.059	0.480	1.011	0.967–1.057	0.625
Gender (male/female)	0.669	0.254–1.764	0.417	0.782	0.290–2.108	0.627
TNM stage (I/II *vs*. III/IV)	2.613	0.902–7.563	0.077	2.698	0.918–7.928	0.071

HR, hazard ratio; CI, confidence interval; lncRNAsig, long non-coding RNA signature; TCGA, The Cancer Genomics Atlas; TNM, tumor node metastasis; FUSCC, Fudan University Shanghai Cancer Center.

Bold type indicates statistical significance.

The Ti-lncRNA signature was analyzed for its associations with the prognostic outcomes of 32 malignancies using the pan-cancer TCGA data as well, of which 18 malignancies demonstrated increased disease risk patients with high signature risk scores compared to those with low risk scores, especially including kidney renal papillary cell carcinoma (KIRP), lung adenocarcinoma (LUAD), and pancreatic adenocarcinoma (PAAD) (**Supplementary Figure S4**).

### Correlations of the Ti-lncRNA Signature With Clinicopathological Features, Genetic Mutations, Hypoxia, CAF, and TAM in TME

**Figure 5:**
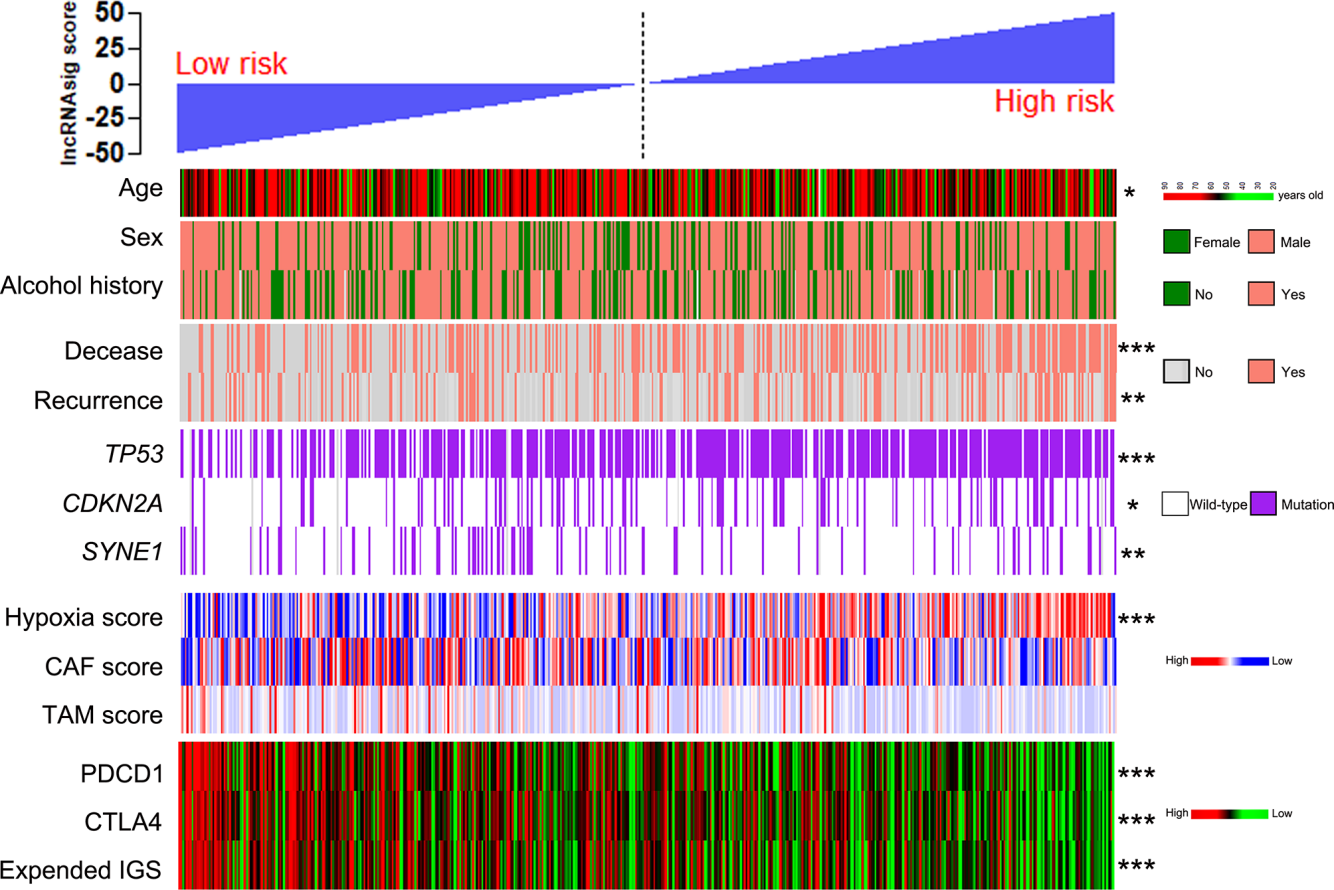
Patients from TCGA cohort with high and low Ti-lncRNA scores were ranked by clinicopathological factors, genetic mutations, the hypoxia, CAF, and TAM scores, PDCD1 and CTLA4 expressions, and the expanded IGS score. *p < 0.05, **p < 0.01, ***p < 0.001. lncRNA, long non-coding RNA; Ti-lncRNA, tumor-infiltrating immune-related lncRNA; TCGA, The Cancer Genome Atlas; CAF, cancer-associated fibroblast; TAM, tumor-associated macrophage; IGS, immune gene signature.

Using the cohort data from TCGA, we analyzed the correlations of the Ti-lncRNA signature with the clinicopathological features, genetic mutations, hypoxia, CAF, and TAM in TME. The Ti-lncRNA signature showed no statistical associations with gender, alcohol history, HPV status, TNM stage, and histological grade, except for age. In the analyses of the factors affecting the Ti-lncRNA signature, hypoxia (*p* < 0.001) and *TP53* (*p* < 0.001) and *CDKN2A* (*p* = 0.048) mutations showed significant associations with a high signature score, while *SYNE1* mutation (*p* = 0.003) occurred more frequently in patients with low risk scores than in those with high risk scores (**Figure 5** and **Supplementary Table S3**). CAF and TAM failed to have an impact on the Ti-lncRNA signature. After adjusting for age, sex, alcohol history, hypoxia, and *TP53*, *CDKN2A*, and *SYNE1* mutations, the multivariate logistic regression analysis showed that hypoxia (*p* < 0.001) and *TP53* mutation (*p* = 0.001) were independent risk factors for patients with a high signature score, whereas *SYNE1* mutation was an independent protective factor (*p* = 0.004) (**Supplementary Table S3**).

### The Ti-lncRNA Signature and Immunotherapy Response

**Figure 6:**
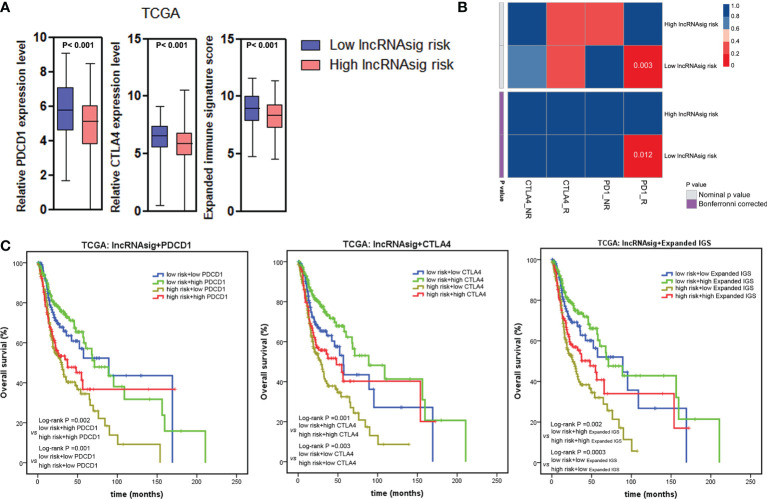
Ti-lncRNA signature and immunotherapy response. **(A)** Associations of the signature risk score with the expression of PDCD1 and CTLA4 and the expanded IGS score. **(B)** Sub-map analyses of the Ti-lncRNA signature in melanoma patients undergoing blockade therapy of PD-1 and CTLA4. **(C)** Kaplan–Meier survival curves of OS among four patient groups stratified by the Ti-lncRNA signature, PDCD1 and CTLA-4 expressions, and the expanded IGS scores. lncRNA, long non-coding RNA; Ti-lncRNA, tumor-infiltrating immune-related lncRNA; TCGA, The Cancer Genome Atlas; CAF, cancer-associated fibroblast; TAM, tumor-associated macrophage; IGS, immune gene signature; OS, overall survival.

To investigate the predictive effect of the Ti-lncRNA signature on blockade therapy of immune checkpoints, we initially analyzed the relationship between the signature score and the expression of immune checkpoints in HNSCC. As shown in **Figures 5**, **6A**, the signature score showed a negative correlation with the expressions of PDCD1 and CTLA4 (**Supplementary Table S3**). We also utilized an expanded IGS consisting of 18 genes that can be predictive of the response to anti-PD-1 therapy for HNSCC in order to verify the value of the Ti-lncRNA signature on the evaluation of immunotherapy response (41). Consistently, HNSCC patients with low signature scores demonstrated increased expended IGS scores, suggesting an effective response to anti-PD-1 therapy (**Figures 5**, **6A** and **Supplementary Table S3**). Moreover, using available data of melanoma patients undergoing immunotherapy, it was found that anti-PD-1 therapy tended to achieve good responses in patients with low Ti-lncRNA signature scores (**Figure 6B**).

Integrating the Ti-lncRNA signature and immune checkpoints to analyze their impacts on the prognoses of patients, we found that a high signature score was significantly associated with a reduced OS independent of the level of PDCD1 or CTLA4 or the expanded IGS score (**Figure 6C**). Patients with the combination of a high signature risk score and a low level of PDCD1 or CTLA4 or expanded IGS score had the worst survival prognosis, while those with a low risk score and a high level of the immune factor had extended survival time.

## Discussion

In addition to the existing FDA-approved PD-1 inhibitors, nivolumab and pembrolizumab, emerging agents targeting PD-1 and CTLA4 are under ongoing clinical trials for HNSCC patients (3). So far, limited knowledge on tumor-infiltrating immune cells has confined the ability to effectively predict the effects of immunotherapy. Although some studies have reported that lncRNAs in immune cells play critical roles in tumor cell–immune cell interactions (12, 13), the roles of lncRNAs remain unclear in tumor-infiltrating immune cells of HNSCC. Due to the temporal and spatial specificity of the expressions of lncRNAs in human cells, tissues, and organs, alterations in their expressions can be well predictive of the state of cells and their response to stimuli (12, 42, 43). The present study aimed to explore the characteristics of lncRNA expressions in HNSCC-infiltrating immune cells in order to screen out a lncRNA signature that can clinically reflect survival prognoses and effectively predict response to therapy targeting immune checkpoints for HNSCC.

Therefore, to our knowledge, this is the first time a novel model was built for the lncRNA expression patterns of tumor-infiltrating immune cells in HNSCC. Specific expressions of lncRNAs were initially evaluated in 19 types of immune cells, and then the top 10% lncRNAs were selected in each immune cell type. Each lncRNA was further analyzed for its cell and tissue specificity index. Comparison analyses of the lncRNAs between 19 immune cell types and HNSCC cell lines identified the upregulated lncRNAs in immune cells, which were then validated in HNSCC patients for their prognostic associations in order to develop a prognostic lncRNA model of immune cells for HNSCC. As a result, a signature of nine lncRNAs was discovered in the training cohort of TCGA and validated in the test cohort.

The Ti-lncRNA signature was demonstrated to be a prognostic predictor for HNSCC patients among the TCGA, FUSCC, GSE41613, and GSE42743 cohorts. A high signature score was an independent risk factor for both death and recurrence in HNSCC patients. In addition, the signature was confirmed to be associated with the prognoses of patients for multiple types of cancers in the pan-cancer analyses, suggesting its efficacy as a prognostic factor for cancer. The significant correlations of the Ti-lncRNA signature with poor prognoses may be attributed to the alterations of the expressions of Ti-lncRNAs associated with immunosuppressive TME, which could either affect the activated state of tumor-infiltrating immune cells or receive tumor–stromal crosstalk signaling from aggressive cancer cells.

It is well known that the intricate interaction between tumor cells and stromal cells within the TME contributes to immune evasion and immunotherapy resistance (44–46). In our study, a high signature score was found to be positively correlated with mutations in *TP53* and *CDKN2A*, the most frequently altered tumor suppressor genes in HNSCC. In addition to its significant associations with shortened survival time and resistance to radiotherapy and chemotherapy (47), *TP53* mutation has been recently reported by Zhang et al. to be indicative of poor response to immunotherapy in HNSCC (48), which supports the signature as a potential predictor for prognoses and immunotherapy response. Immunosuppressive TME is characterized by enriched CAFs, TAMs, T regulatory cells, myeloid-derived suppressor cells, and hypoxia, leading to tumor progression and reduced responses to pembrolizumab and nivolumab (49). Therefore, we explored the correlations of the signature score with the CAF, TAM, and hypoxia scores as well. Patients with high signature scores tended to stay at high hypoxia status. There was no statistical association of the signature score with the CAF and TAM scores in our study. It is interesting to find that *TP53* mutation in tumor cells and hypoxia within the TME can affect the signature score independently. A series of studies have revealed that *TP53* mutation confers an immunosuppressive phenotype in multiple tumors (50–52). Hypoxia also plays a pivotal role in immunosuppressive effect in a variety of ways, such as reducing the activities of cytotoxic T cells and natural killer (NK) cells, increasing the release of immunosuppressive cytokines, and inducing the expressions of immune checkpoint inhibitors (53, 54). These outcomes may suggest that tumor cells harboring *TP53* mutations alter the lncRNA expression patterns through crosstalk with immune cells and that hypoxia significantly induces the expressions of the signature lncRNAs in immune cells.

The signature score has been confirmed to be negatively correlated with the expressions of PDCD1 and CTLA4, suggesting that the signature score can provide evidence for determining blockade therapy of immune checkpoints. Ayers et al. described the expanded IGS consisting of 18 genes, and a high expanded IGS was associated with the clinical response to PD-1 blockade for HNSCC patients in a previous study (41). The correlation of a low signature score with a high expanded IGS further verified its predictive effect for anti-PD-1 response. Consistent with the above results, patients with low signature scores showed clinical response to anti-PD-1 therapy in melanoma. Thus, our study reveals that a low signature score is indicative of response to blockade of immune checkpoints, while a high score means increased risk of therapeutic resistance. This finding can be explained by the following aspects: on one hand, a majority of the activated immune cell signaling pathways were enriched in patients with low signature scores, as described in the context; on the other hand, PDCD1 and CTLA4 exhibited high expressions in patients with low signature scores.

Finally, we have to stress that some limitations exist in the present study. Because our clinical trials of anti-PD-1 therapy are ongoing, it is not available for us to evaluate the predictive effect of the Ti-lncRNA signature on therapeutic response to immunotherapy. Although the signature is a potent indicator of immunotherapy response, the molecular mechanisms of the nine lncRNAs in immunosuppressive TME remain unclear. Therefore, the next step is to validate the predictive value of this signature in our patients undergoing immunotherapy; an RNA scope will be performed to determine the localization and distribution of the nine Ti-lncRNAs in immune cells. Moreover, the Ti-lncRNAs specifically localized in cytotoxic T lymphocytes will be selected with priority according to the results of the RNA scope, which will be further investigated for their biological functions in the transformation between immunoactivation and immunosuppression and the crosstalk between tumor cells and immune cells.

In summary, the Ti-lncRNA signature was identified as a prognostic factor independent of the TNM stage, PDCD1, CTLA4, and the expanded IGS, which may become a potential clinical indicator of therapeutic response to anti-PD-1/PD-L1 and anti-CTLA4 therapies.

## Data Availability Statement

The datasets presented in this study can be found in online repositories. The names of the repository/repositories and accession number(s) can be found in the article/supplementary material.

## Ethics Statement

The studies involving human participants were reviewed and approved by Medical Ethics Committee of Fudan University Shanghai Cancer Center. The patients/participants provided their written informed consent to participate in this study.

## Author Contributions

YW, QJ, CD, and BM designed the study. BM, HJ, YL, and TL were responsible for performing experiments. BM and HJ performed data analyses. WX and XW contributed to the collection of surgical samples and clinical information and the data preparation. BM and CD wrote the manuscript. YW and QJ revised the paper. All authors contributed to the article and approved the submitted version.

## Funding

The study was supported by the National Natural Science Foundation of China (82072951 to YW), the Science and Technology Commission of Shanghai Municipality (19411966600 to YW), and the Shanghai Anticancer Association (SACA-AX106 to YW and SACA-CY19B01 to BM).

## Conflict of Interest

The authors declare that the research was conducted in the absence of any commercial or financial relationships that could be construed as a potential conflict of interest.

The reviewer LT declared a shared affiliation with the authors to the handling editor at the time of the review.

## Publisher’s Note

All claims expressed in this article are solely those of the authors and do not necessarily represent those of their affiliated organizations, or those of the publisher, the editors and the reviewers. Any product that may be evaluated in this article, or claim that may be made by its manufacturer, is not guaranteed or endorsed by the publisher.

## Supplementary Material

Supplementary Figure 1 Heatmap of differential expression pattern observed in 19 immune cell types.

Supplementary Figure 2 The immune cell specificity of the nine lncRNAs.

Supplementary Figure 3 The Ti-lncRNA signature’s associations with prognostic outcomes of 32 malignancies. **(A)** Hazards ratios (HR) with 95% confidence interval (CI) of the signature were calculated in the analyses of OS in 32 malignancies by using the pan-cancer TCGA data; **(B-D)** OS difference of the signature risk score was analyzed in kidney renal papillary cell carcinoma (KIRP) **(B)**, lung adenocarcinoma (LUAD) **(C)** and pancreatic adenocarcinoma (PAAD) **(D)**.

Supplementary Table 1 Primer sequence of the Ti-lncRNA signature genes, and hypoxia and CAF signature.

Supplementary Table 2 LncRNA landscape of HNSCC-infiltrating immune cells.

Supplementary Table 3 Tissue specificity index (TSI) scores of the nine lncRNAs included in our signature and associations of the Ti-lncRNA signature with clinicopathological and molecular factors.
