# Supplementary material for: Tumor-Infiltrating Immune-Related Long Non-Coding RNAs Indicate Prognoses and Response to PD-1 Blockade in Head and Neck Squamous Cell Carcinoma
Source: Front Immunol. 2021 Oct 19;12:692079. doi: 10.3389/fimmu.2021.692079 (PMC8562720; doi:10.3389/fimmu.2021.692079)
Supplement: Supplementary Figure 1 — Heatmap of differential expression pattern observed in 19 immune cell types. [file DataSheet_1.pdf]

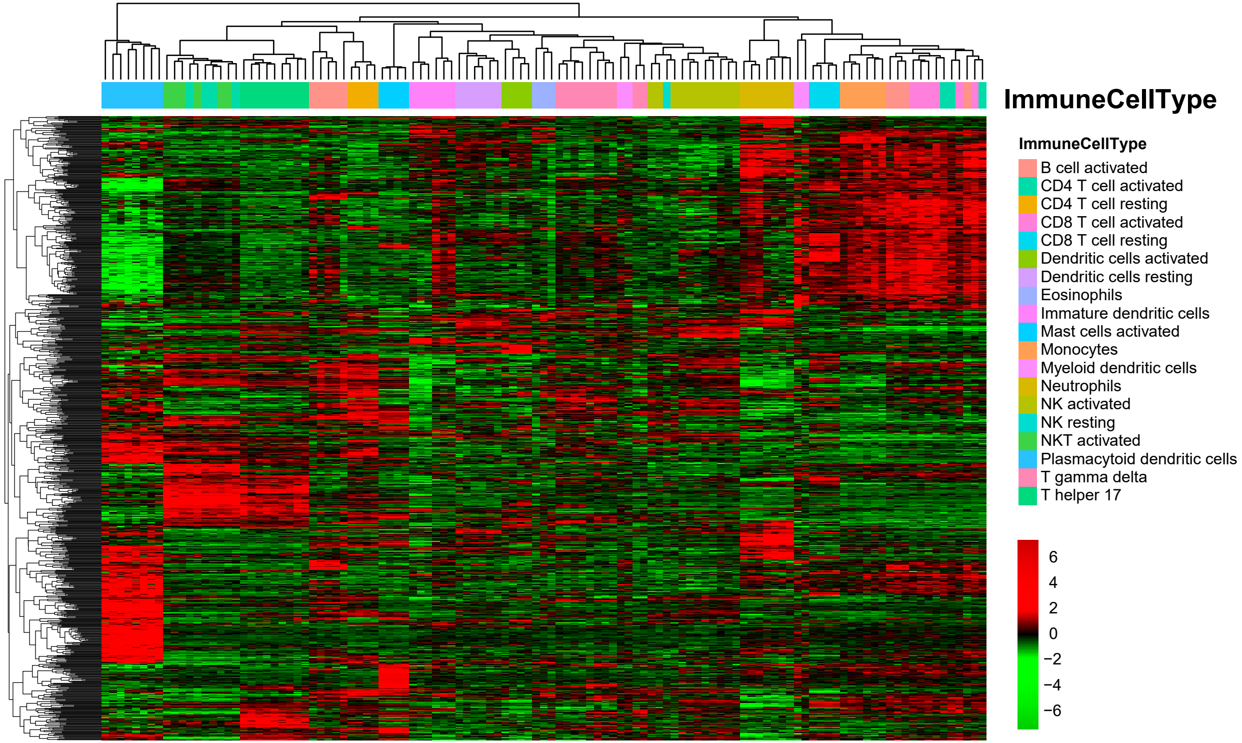
ImmuneCellType
ImmuneCellType
B cell activated
CD4 T cell activated
CD4 T cell resting
CD8 T cell activated
CD8 T cell resting
Dendritic cells activated
Dendritic cells resting
Eosinophils
Immature dendritic cells
Mast cells activated
Monocytes
Myeloid dendritic cells
Neutrophils
NK activated
NK resting
NKT activated
Plasmacytoid dendritic cells
T gamma delta
T helper 17
6
4
2
0
−2
−4
−6
