# Supplementary figures and images for: Tumor-Infiltrating Immune-Related Long Non-Coding RNAs Indicate Prognoses and Response to PD-1 Blockade in Head and Neck Squamous Cell Carcinoma
Source: Front Immunol. 2021 Oct 19;12:692079. doi: 10.3389/fimmu.2021.692079 (PMC8562720; doi:10.3389/fimmu.2021.692079)

TSPOAP1-AS1

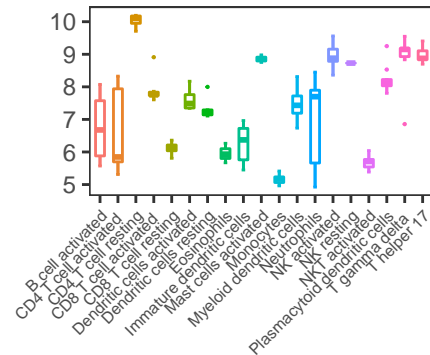

RASSF1-AS1

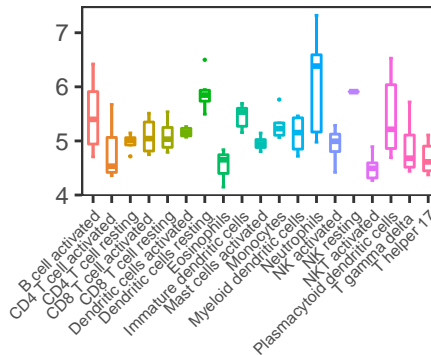

ENSG00000262089

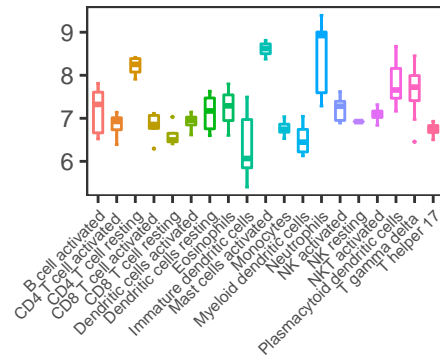

NDUFB2-AS1

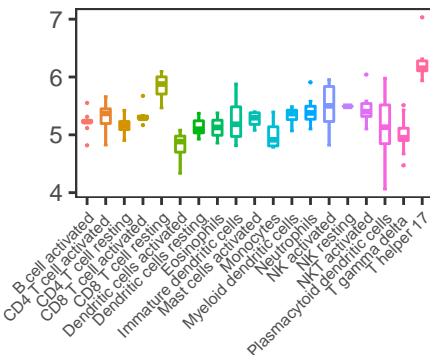

MIR124-1HG

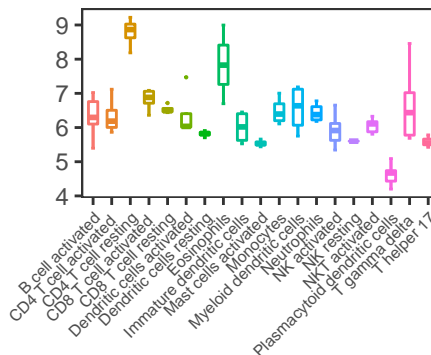

ENSG00000261888

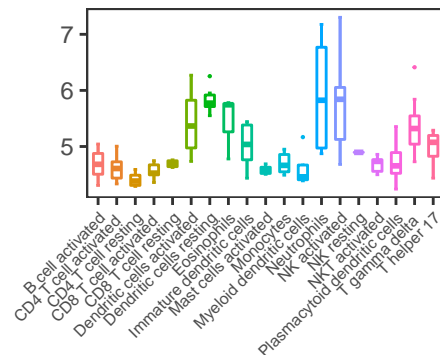

LINC01281

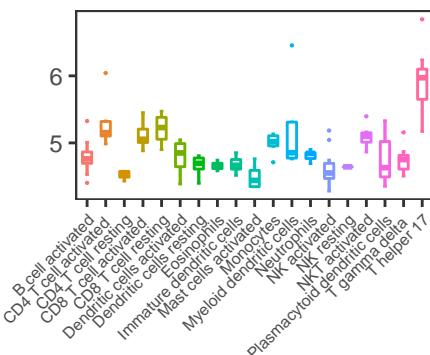

LCT-AS1

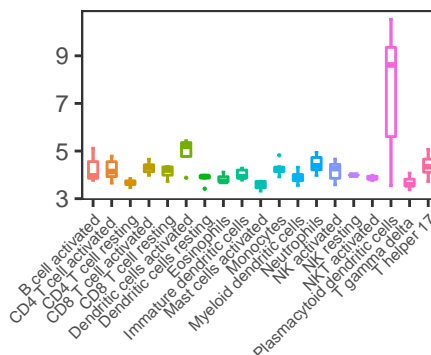

ENSG00000260244

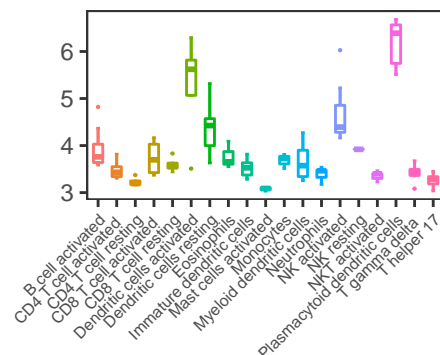

Supplement: Supplementary Figure 2 — The immune cell specificity of the nine lncRNAs. [file DataSheet_2.pdf]

A

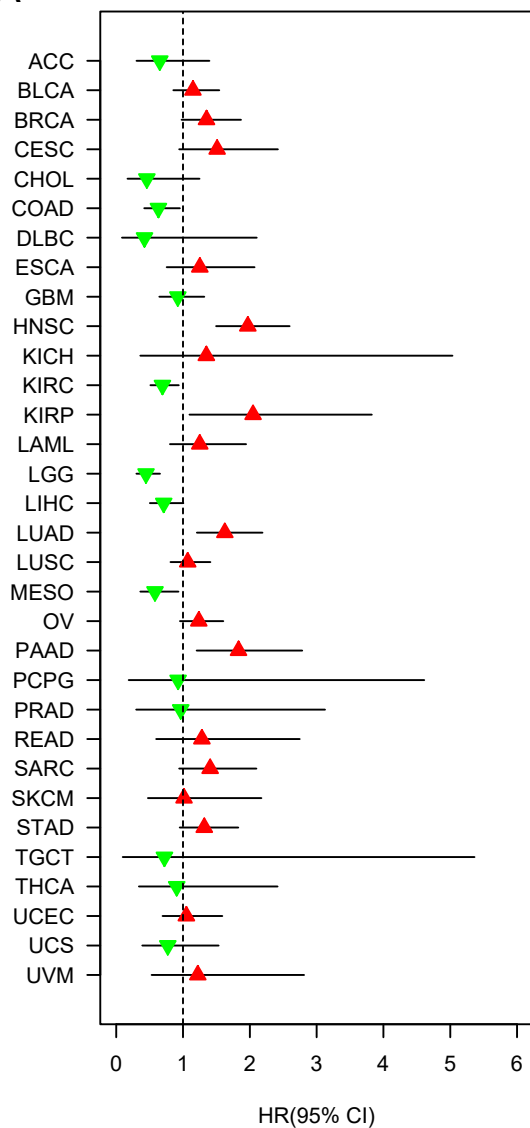

B

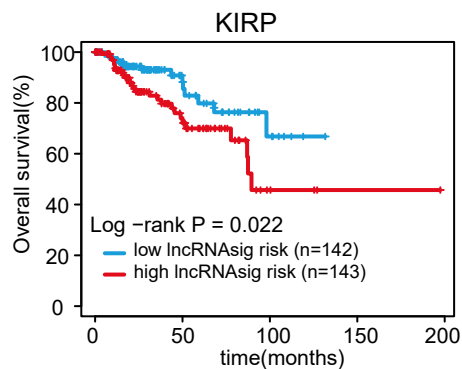

C

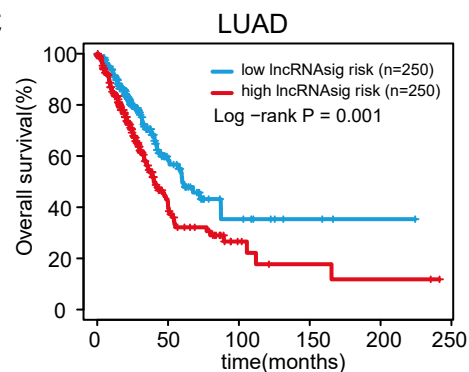

D

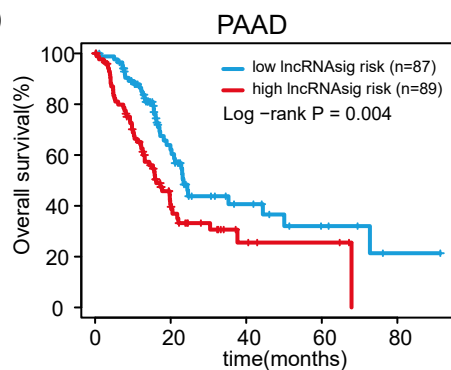

Supplement: Supplementary Figure 3 — The Ti-lncRNA signature’s associations with prognostic outcomes of 32 malignancies. (A) Hazards ratios (HR) with 95% confidence interval (CI) of the signature were calculated in the analyses of OS in 32 malignancies by using the pan-cancer TCGA data; (B-D) OS difference of the signature risk score was analyzed in kidney renal papillary cell carcinoma (KIRP) (B), lung adenocarcinoma (LUAD) (C) and pancreatic adenocarcinoma (PAAD) (D). [file DataSheet_3.pdf]
